# Soybean productivity can be enhanced by understanding rhizosphere microbiota: evidence from metagenomics analysis from diverse agroecosystems

**DOI:** 10.1186/s40168-025-02104-y

**Published:** 2025-04-26

**Authors:** Honglei Ren, Huilong Hong, Bire Zha, Sobhi F. Lamlom, Hongmei Qiu, Yongqiang Cao, Rujian Sun, Haorang Wang, Junkui Ma, Hengbin Zhang, Liping Sun, Qing Yang, Changjun Zhou, Xiulin Liu, Xueyang Wang, Chunlei Zhang, Fengyi Zhang, Kezhen Zhao, Rongqiang Yuan, Ahmed M. Abdelghany, Bixian Zhang, Yuhong Zheng, Jiajun Wang, Wencheng Lu

**Affiliations:** 1 Soybean Research Institute of Heilongjiang Academy of Agriculture Sciences, Harbin, 150086 China; 2 National Key Facility for Crop Gene Resources and Genetic Improvement, Institute of Crop Sciences, Chinese Academy of Agricultural Sciences, Beijing, 100081 China; 3 College of Modern Agriculture and Ecological Environmentofaq , Heilongjiang University, Harbin, 150006 China; 4 Plant Production Department, Faculty of Agriculture Saba Basha, Alexandria University, Alexandria, 21531 Egypt; 5 Jilin Academy of Agriculture Sciences (Northeast Agricultural Research Center of China), Changchun, 130033 China; 6 Crop Research Institute of Liaoning Academy of Agriculture Sciences, Shenyang, 110161 China; 7 Hulunbuir Institute of Agriculture and Animal Husbandry, Hulunbuir, 021000 China; 8 Jiangsu Xuhuai Regional Institute of Agricultural Sciences, Xuzhou, 221131 China; 9 Shanxi Agricultural University/ Shanxi Academy of Agricultural Sciences, the Industrial Crop Institute, Taiyuan, 030031 China; 10 Xinjiang Academy of Agricultural and Reclamation Science, Shihezi, 832000 China; 11 Jiangxi Academy of Agricultural Sciences, Nanchang, 330200 China; 12 Institute of Cereal and Oil Crops, Hebei Academy of Agricultural and Forestry Sciences, Shijiazhuang, 050035 China; 13 Daqing Branch of Heilongjiang Academy of Agricultural Sciences, Daqing, 163316 China; 14 Crop Science Department, Faculty of Agriculture, Damanhour University, Damanhur, 22516 Egypt; 15 Institute of Biotechnology of Heilongjiang Academy of Agricultural Sciences, Harbin, 150086 China; 16 Heihe Branch Institute of Heilongjiang Academy of Agricultural Sciences, Heihe, 164300 China

**Keywords:** Soybean (*Glycine max*), Rhizosphere microbiota, Metagenomic sequencing, Co-occurrence networks

## Abstract

**Background:**

Microbial communities associated with roots play a crucial role in the growth and health of plants and are constantly influenced by plant development and alterations in the soil environment. Despite extensive rhizosphere microbiome research, studies examining multi-kingdom microbial variation across large-scale agricultural gradients remain limited.

**Results:**

This study investigates the rhizosphere microbial communities associated with soybean across 13 diverse geographical locations in China. Using high-throughput shotgun metagenomic sequencing on the BGISEQ T7 platform with 10 GB per sample, we identified a total of 43,337 microbial species encompassing bacteria, archaea, fungi, and viruses. Our analysis revealed significant site-specific variations in microbial diversity and community composition, underscoring the influence of local environmental factors on microbial ecology. Principal coordinate analysis (PCoA) indicated distinct clustering patterns of microbial communities, reflecting the unique environmental conditions and agricultural practices of each location. Network analysis identified 556 hub microbial taxa significantly correlated with soybean yield traits, with bacteria showing the strongest associations. These key microorganisms were found to be involved in critical nutrient cycling pathways, particularly in carbon oxidation, nitrogen fixation, phosphorus solubilization, and sulfur metabolism. Our findings demonstrate the pivotal roles of specific microbial taxa in enhancing nutrient cycling, promoting plant health, and improving soybean yield, with significant positive correlations (*r* = 0.5, *p* = 0.039) between microbial diversity and seed yield.

**Conclusion:**

This study provides a comprehensive understanding of the diversity and functional potential of rhizosphere microbiota in enhancing soybean productivity. The findings underscore the importance of integrating microbial community dynamics into crop management strategies to optimize nutrient cycling, plant health, and yield. While this study identifies key microbial taxa with potential functional roles, future research should focus on isolating and validating these microorganisms for their bioremediation and biofertilization activities under field conditions. This will provide actionable insights for developing microbial-based agricultural interventions to improve crop resilience and sustainability.

Video Abstract

**Supplementary Information:**

The online version contains supplementary material available at 10.1186/s40168-025-02104-y.

## Introduction

The root-associated microbiome is known as the “second genome” of plants and plays diverse roles in plant growth, development, and health [1]. The compartments associated with the roots such as the rhizosphere offer distinct environments for microbial colonization, leading to significant variations in taxonomy and function compared with the surrounding soil [2]. Nevertheless, distinct root-associated microhabitats are characterized by their unique composition of mineral nutrients, microbial communities, and metabolic profiles [3]. The magnitude of the impact varies depending on individual biological and chemical factors as well as environmental conditions [4]. The rhizosphere microbiome dynamics of soybean (*Glycine max*), a globally cultivated leguminous crop, have been extensively characterized through systematic investigations [5, 6]. Compared with those in the surrounding soil, the bacterial communities in the rhizosphere of soybean plants are less varied [7]. This suggests a specific selection of microorganisms specialized in nitrogen, iron, phosphorus, and potassium metabolism [8, 9].

Extensive research has revealed that the rhizosphere microbiome exhibits a unique taxonomic and functional composition that markedly diverges from bulk soil microbial communities [10]. Furthermore, plant community diversity, species, genotype, developmental stage, root shape, and exudation have all been implicated in the influence of plant roots on soil microorganisms [11]. Because rhizosphere composition varies according to the plant’s interactions with insects, soil microorganisms, and other plants, as well as its species, genotype, and developmental stage, rhizodeposition in particular has a more direct impact on soil bacteria [12]. These findings indicate that plants actively alter rhizosphere microbial communities in a genotype-specific manner, likely through the release of metabolites [13]. Plant uptake in the rhizosphere influences water and mineral concentrations. Research has demonstrated that the contents of some nutrients, such as nitrogen, phosphorus, and potassium, decrease from the bulk soil to the surface of the roots [14]. Moreover, microbial activity significantly affects the availability of minerals in the rhizosphere [4]. The distinct biological and chemical conditions in the compartments linked to the roots can directly influence plant growth and perhaps reveal the physiological state of the host plant. Therefore, a comprehensive understanding of the surrounding conditions is essential to promote strong plant growth and maintain consistent crop production, especially when faced with environmental changes.

Rhizosphere microorganisms mitigate environmental stress and pressure, contribute to the preservation or enhancement of environmental sustainability, and increase overall ecological conditions [15, 16]. The maintenance of diverse and functionally redundant rhizosphere bacterial communities, rather than merely increased bacterial abundance, is crucial for the long-term viability of soybean cultivation, as these communities can enhance plant resilience under various environmental stresses [17, 18]. The microbiomes associated with plant roots significantly fluctuate and are strongly influenced by the growth and maturation of plants [1, 19, 20]. The plant primarily influences microbial growth through the action of root exudates, demonstrating its capacity to actively alter its microbiome throughout its whole existence [21]. This property could have significant effects on the acquisition of nutrients by plants, as indicated by the temporal complementarity of nitrogen usage efficiency between roots and microorganisms [22]. The rhizosphere microbial community structure of each legume plant is distinct [23]. Consequently, there are variations in the composition of rhizosphere microbial communities across several soybean genotypes [24].

Research indicates that the rhizosphere fungal and bacterial communities of various plants, including *Arabidopsis*, *Medicago*, maize, pea, wheat, and sugar beet, fluctuate in accordance with a plant developmental gradient [25, 26]. The investigations indicated that plant microbial communities alter in response to plant development, and they failed to identify how or which bacteria contribute to the observed alterations. Micallef et al. [26] utilized denaturing gradient gel electrophoresis analysis to observe that the microbial communities in the *Arabidopsis* rhizosphere varied with plant development, noting that these communities were more distinct from the bulk soil during early plant development, with this distinction diminishing as the plant aged. The microbial communities in the soybean rhizosphere were affected by plant development, with early reproductive growth stages yielding more complex microbial communities compared to late-stage soybean plants [19, 27]. An evaluation of the microbial community structure throughout plant development, concentrating on its constituent members, is necessary. The recent characterization of the *Arabidopsis thaliana* core microbiome serves as a tool to elucidate the plant’s impact on the rhizosphere microbiome during various developmental phases [28, 29].

Advanced omics approaches (metagenomics, meta-transcriptomics, and RNA-seq) have revolutionized our understanding of microbial communities, providing unprecedented insights into their diversity, functional potential, and ecological interactions in both environmental and host-associated systems [30, 31]. While these methodologies offer complementary approaches to understanding microbial ecosystems, our study focuses specifically on metagenome analysis to characterize the bacterial population structure. Moreover, because of the large amount of information generated by next-generation sequencing (NGS), plant RNA-seq datasets that were originally generated to study the host transcriptome may be a novel resource for studying the plant-associated microbiota [32]. While substantial research has examined rhizosphere microbiomes in controlled environments or limited geographical contexts [33, 34], significant knowledge gaps remain in understanding how these communities vary across large-scale geographic gradients, particularly in major agricultural regions [35]. Most previous studies have focused on single locations or compared only a few sites [36, 37], limiting our understanding of how spatial heterogeneity and diverse agricultural practices influence rhizosphere microbial communities at a regional scale [38]. Additionally, while individual components of the microbiome (bacteria, fungi, or archaea) have been well-studied [39, 40], comprehensive analyses incorporating all microbial kingdoms simultaneously across diverse agricultural landscapes remain scarce [41]. While metagenomic analyses of plant-associated microbiomes have revealed novel phylogenetic and functional insights, the predominant focus on healthy plant systems [42] limits our understanding of the factors governing beneficial plant–microbe interactions and their dynamic relationships. The primary objective of this study is to investigate the rhizosphere microbial communities associated with soybean crops across 13 diverse geographical locations in China and to explore their influence on nutrient cycling and soybean yield. Specifically, the study aims to profile the microbial communities, including bacteria, archaea, fungi, and viruses, present in the rhizosphere using high-throughput metagenomic sequencing. It seeks to assess site-specific variations in microbial diversity and community composition about local environmental factors and agricultural practices.

## Materials and methods

### Plant materials, site selection, and experimental establishment

The research comprehensively investigated soybean production across China by strategically selecting 13 study sites representing major soybean-producing regions. The site selection process was based on three primary criteria: (1) historical importance in soybean production, (2) representation of distinct agroecological zones, and (3) availability of long-term agricultural management records. The selected sites spanned a remarkable latitudinal gradient from 28°33′N to 47°59′N and a longitudinal gradient from 86°0′E to 129°34′E, capturing significant environmental variability across China’s agricultural landscape.

In 2023, primary local soybean varieties were selected for cultivation in areas no less than 667 m^2^ (1/15 ha) per site, with sowing and harvesting dates adhering to local agricultural customs. The specific varieties and their precise locations were as follows: In Heilongjiang province, Heinong551 was located in Harbin city (126°51′10.80″E, 45°50′31.20″N), Heinong 531 in Daqing city (125°19′16.59″E, 46°62′5.31″N), and Mudou10 in Mudanjiang city (129°34′48.00″E, 44°30′50.40″N). In Liaoning province, Liaodou32 was cultivated in Shenyang city (123°33′10.80″E, 41°49′26.40″N), while Zhongji602 was planted in Gongzhuling city, Jilin province (124°48′25.20″E, 43°30′50.40″N). Shanxi province was represented by Fendou 93, located in Fenyang city (111°47′24.00″E, 37°14′42.00″N). In Shandong province, Qihuang34 was planted in Jinan city (117°4′44.40″E, 36°42′25.20″N), and in Hebei province, Jidou17 was cultivated in Shijiazhuang city (114°26′52.80″E, 38°3′25.20″N). Gansu province was represented by Zhonghuang 35, located in Lanzhou city (103°41′16.80″E, 36°6′3.60″N). Mengdou1137 was planted in Zhalantun city, Inner Mongolia (122°42′32.40″E, 47°59′52.80″N), and Xindadou1 was established in Shihezi city, Xinjiang (86°0′21.60″E, 44°18′28.80″N). The southern regions were represented by Gandou19 in Nanchang city, Jiangxi province (115°56′31.20″E, 28°33′28.80″N), and Xudou 18 in Xuzhou city, Jiangsu province (117°23′49.20″E, 34°8′24.00″N). This comprehensive cultivation strategy reflected the diverse climatic and geographical conditions across China’s agricultural landscape, optimizing each region’s specific strengths for soybean production.

For each soybean variety, 10 replicate plots were established at their respective locations, with each plot covering an area of 667 m^2^. The experimental design maintained standardized row spacing and plant density following local agricultural recommendations, ensuring uniformity while respecting regional cultivation practices. The soil sampling approach was designed to ensure representative and reproducible results through a systematic “S” sampling pattern within each plot. This method involved identifying 10 sampling points across each plot, where rhizosphere soil was collected from three adjacent soybean plants at each point, ensuring a consistent and representative strategy. The soil was sampled at a uniform depth of 0–20 cm, within 5 mm of the plant roots, and the three subsamples from each point were thoroughly homogenized to create a composite sample. This rigorous process produced 10 biological replicates per site, resulting in a total of 130 samples across all 13 study sites, enabling a comprehensive investigation of the rhizosphere soil characteristics. To ensure maximum scientific reliability, the research protocol incorporated multiple technical replicates. For each biological sample, three technical replicates were processed for metagenomic analysis, significantly enhancing the robustness of the sequencing data. Immediately after collection, samples were thoroughly mixed and stored at − 80 °C to preserve their integrity for subsequent detailed analysis. To achieve uniformity in the developmental stage and mitigate age-related variations in the rhizosphere microbiome, we sampled all plants at all locations during the blooming phase. This careful approach ensured the highest quality of sample preservation and subsequent scientific investigation.

### Determination of soybean yield traits

Ten plants from 13 soybean varieties across 13 regions were randomly selected for growth and yield measurements. Six traits were measured: plant height (cm), number of pods per plant, number of grains per plant, grain weight per plant (g), 100-grain weight (g), and seed yield (kg/667 m^2^). All the measurements followed the Soybean Germplasm Resource Description Standard and Data Standard [43].

### DNA extraction and quality inspection

A total of 130 soybean rhizosphere soil samples, with 10 samples from each of the 13 regions, were collected for genomic DNA extraction via the Fast DNA SPIN Kit for Soil (MP Biomedicals). From each sample, 300 mg of soil was processed, and the extracted DNA was assessed for quality on 1% agarose gels in 0.5 × TAE buffer and run at 100 V for 15 min. The DNA concentration was quantified via a Qubit® dsDNA Assay Kit and a Qubit® 2.0 fluorometer (Life Technologies). Samples with DNA amounts greater than 1 μg were used to construct sequencing libraries [44].

### Shotgun metagenomic sequencing

DNAs from 130 samples were sequenced on the BGISEQ T7 platform using a pair-end 150 base pair (bp) sequencing strategy, with an average of 10 GB per sample. The quality control process involved the filtering and pruning of 3′ ends of input FASTQ files via fastp software (version 0.12.4) [45]. The generated clean high-quality reads were assembled to generate long contig using megahit (v1.1.3). Open reading frames (ORFs) from each metagenomic sample were predicted using Prodigal (v2.6.3). All sequences from gene sets with a 95% sequence identity (90% coverage) were clustered as the non-redundant gene catalog by the CD-HIT (v4.8.1, http://www.bioinformatics.org/cd-hit/). Non-redundant gene sequences were searched against the NCBI non-redundant protein database using diamond (v0.9.14.115) with *e*-value cutoff of 1e- 5. The KEGG pathway annotation was conducted using diamond against the Kyoto Encyclopedia of Genes and Genomes database (http://www.genome.jp/keeg/) with an *e*-value cutoff of 1e- 5.

### Bioinformatics and statistical analysis

Statistical analyses of the phenotypic data were performed using a two-tailed Student’s *t*-test in GraphPad Prism (version 7.0). Alpha diversity was assessed using the ACE and Chao1 estimators, along with species richness indices, with rarefaction to 10,000 sequences per sample to account for differences in sequencing depth. Statistical comparisons of alpha-diversity metrics between groups were performed using one-way ANOVA with Tukey’s post hoc test and Benjamini–Hochberg correction for multiple comparisons (*p* < 0.05). Beta-diversity analysis was performed using the vegan package (version 2.6–6.1) in R (version 4.4.1). Community dissimilarities were calculated using the Bray–Curtis distance metric, and statistical significance of group differences was tested using PERMANOVA with 999 permutations. The resulting distance matrix was visualized through principal coordinate analysis (PCoA). Hierarchical clustering was conducted based on Bray–Curtis dissimilarities of species abundance data using the unweighted pair group method with arithmetic mean (UPGMA) algorithm, and the resulting dendrogram was used for visualizing community relationships.

Weighted gene co-expression network analysis (WGCNA) was performed using the WGCNA package (v1.72.5) in R [46]. A total of 43,337 mOTUs and 6 phenotypes were included after filtering for features present in at least 20% of samples with a minimum abundance of 0.01%. The soft power threshold (β) was determined using the scale-free topology criterion (*R*^2^ > 0.80) through the pickSoftThreshold function, resulting in *β* = 6. Networks were constructed using signed correlation with a minimum module size of 30 mOTUs and a merge threshold of 0.25. Module eigengenes were calculated as the first principal component of each module. Module membership (MM) was quantified as the correlation between individual mOTU abundance profiles and module eigengenes, while gene significance (GS) was calculated as the absolute correlation between mOTU abundance and phenotypic traits. Hub mOTUs were identified using stringent thresholds of *MM* > 0.8 and *GS* > 0.2, indicating strong intramodular connectivity and phenotype association, respectively. Statistical significance of module-trait relationships was assessed using Pearson correlation with Benjamini–Hochberg correction for multiple testing (*p* < 0.05). Random forest analysis was used to predict the importance of rhizosphere microorganisms in predicting the soybean phenotype. Microorganisms with mean decrease Gini values greater than 0.25 and adjusted *p*-values less than 0.001 were considered significant predictors [47]. Co-occurrence network clustering of hub rhizosphere microorganisms in soybean was constructed by igraph (v2.0.3) of R packages and Gephi (v0.9.7). The clustering heatmap of hub mOTUs within the four modules by ClusterGVis (v0.1.1) of R packages.

### Functional pathway analysis

Metagenomic sequence data were analyzed for functional gene content using multiple cycling-related databases: MCycDB for carbon cycling, NCycDB for nitrogen cycling, PCycDB for phosphorus cycling, and SCycDB for sulfur cycling. Sequences were first quality filtered (Phred score ≥ 20) and aligned against these databases using DIAMOND (version 2.0.15) with an *E*-value cutoff of 1e- 5. KEGG Orthology (KO) assignments were made using a best-hit approach with a minimum identity threshold of 70% and alignment coverage of 70%. Functional pathway abundances were quantified by counting the number of reads mapping to each KO, normalized by the total number of mapped reads per sample to account for sequencing depth differences. The normalized abundances were then aggregated according to their roles in specific steps of the CNPS (carbon, nitrogen, phosphorus, and sulfur) cycling pathways.

To identify significant associations between microbial taxa (mOTUs) and functional genes, Pearson correlation analyses were performed between normalized mOTU abundances and KO abundances. Correlations were considered significant at |r|> 0.7 and *p* < 0.001, with *p*-values adjusted for multiple testing using the Benjamini–Hochberg method. Functional enrichment of specific pathway steps was assessed by comparing the proportion of significantly correlated KOs within each pathway step to the background distribution of all KOs in that pathway using Fisher’s exact test (*p* < 0.05). The functional pathways were categorized into distinct steps: carbon cycle (steps 1–8, including organic carbon oxidation, carbon fixation, fermentation, metabolism, and methanotrophy), nitrogen cycle (steps 1–8, including nitrogen fixation, nitrate reduction, nitrite reduction, nitric oxide reduction, and nitrite ammonification), phosphorus cycle (steps 1–4, including inorganic P solubilization, organic P mineralization, and polyphosphate degradation), and sulfur cycle (steps 3–9, including sulfur oxidation, sulfate reduction, sulfite reduction, and thiosulfate disproportionation). Visualization of the correlation networks between mOTUs and KOs was performed using Cytoscape (version 3.9.1).

## Results

### Geographical distribution of the study sites and predominant microbial communities

**Fig. 1:**
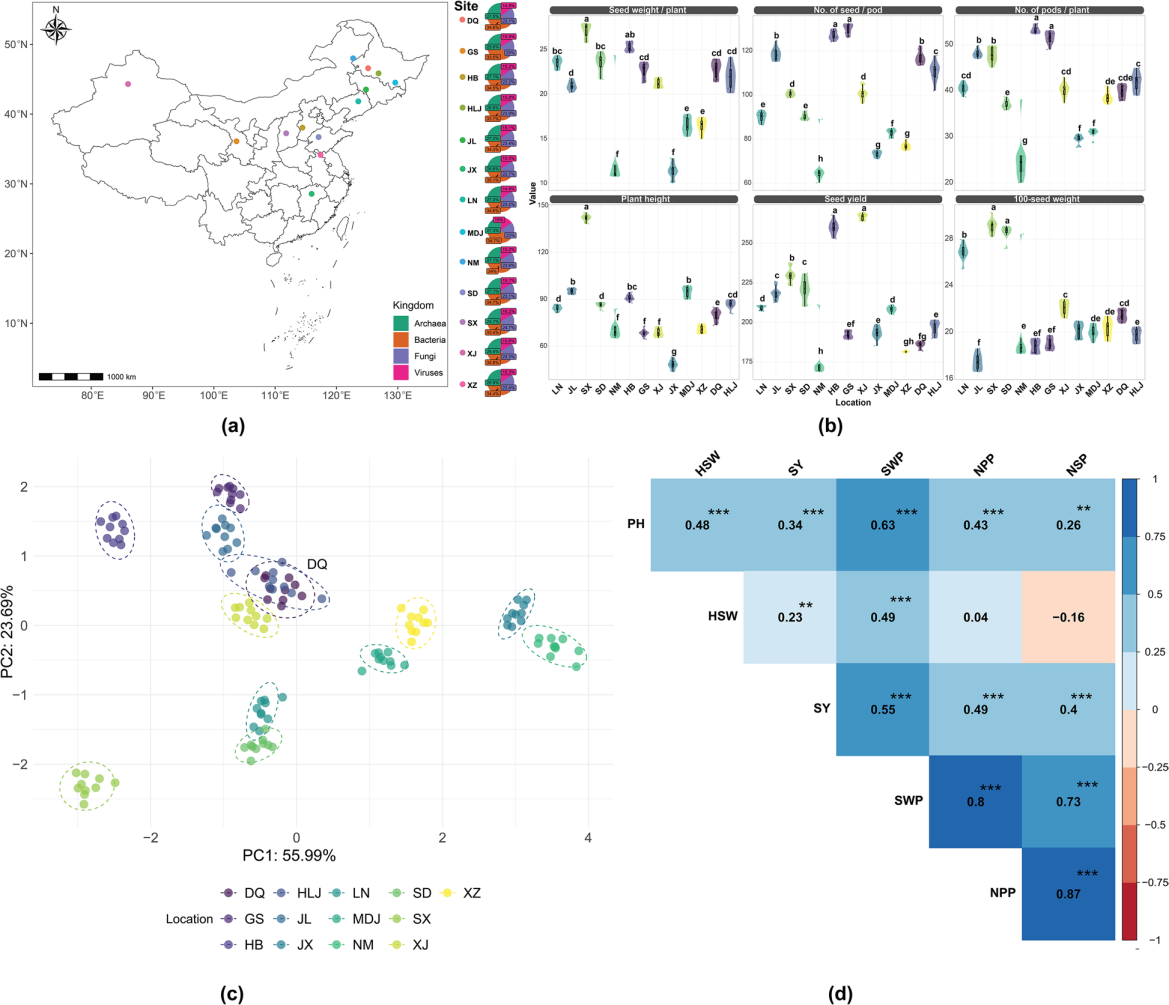
Geographic and trait variation analysis of soybean across China reveals distinct regional patterns and trait correlations. **a** Distribution of 13 sampling sites across China, with colored dots indicating the dominant microbial kingdom at each location. The sites span diverse geographical regions from northeastern to southern China, showing varied microbial compositions (archaea, bacteria, fungi, and viruses). **b** Violin plots demonstrating significant variation in six agronomic traits across locations. The plots reveal distinct regional adaptations, with different letters indicating significant differences between sites (*p* < 0.05). Notable variations are observed in seed weight per plant and plant height, suggesting strong environmental influences on these traits. **c** Principal component analysis (PCA) reveals clear spatial clustering of soybean traits by location, with PC1 (55.99%) and PC2 (23.69%) explaining a substantial 79.68% of total variance. The distinct clustering patterns (highlighted by 95% confidence ellipses) indicate location-specific trait combinations, particularly evident for sites DQ and HLJ. **d** Correlation matrix highlights strong positive relationships between yield-related traits, particularly among SWP, NPP, and SY (correlation coefficients > 0.7, ***p < 0.001). **p < 0.01, ***p < 0.001. NSP, number of seeds per pod; NPP, number of pods per plant (NPP); SWP, seed weight per plant; PH, plant height; HSW, 100-seed weight; SY, seed yield

To elucidate the impact of rhizosphere soil microorganisms on soybean yield and growth, soybean samples and rhizosphere soils were systematically collected from 13 diverse locations across China (Fig. 1). The geographical distributions of these sites, which span a range of latitudes and longitudes to encompass various environmental conditions, are depicted in Fig. 1a. Each site is categorized on the basis of the predominant microbial kingdom present: Archaea, Bacteria, Fungi, and viruses. The prevalence of these microbial communities varied significantly among the locations, with archaea ranging from 25.6% (GS) to 27.5% (DQ), bacteria ranging from 33.4% (SX) to 35.1% (JX), fungi ranging from 22.7% (JX) to 25% (GS), and viruses ranging from 14.8% (DQ and LN) to 16.2% (SX). Notably, bacteria constituted the highest percentage of the microbial community at the JX site (35.1%), whereas viruses were consistently found in lower percentages across all locations. These spatial variations in microbial community composition suggest significant interactions between local environmental factors and microbial dynamics, which could influence the agronomic performance of soybean.

To investigate the geographical variation in soybean agronomic traits, we assessed 6 key traits across 13 diverse locations (Fig. 1b). Our analysis revealed significant geographical variation in all measured traits (*p* < 0.05). Plant height (PH) significantly differed across locations (*p* < 0.05), ranging from 48.23 cm in JX to 142.16 cm in SX. The number of pods per plant (NPP) also showed significant location-dependent differences, with GS and HB having the highest pod numbers (51.58 and 53.30, respectively), which were statistically indistinguishable from each other but significantly greater than those of the other locations (*p* < 0.05). The number of seeds per pod (NSP) varied significantly, with GS producing the greatest number (130.01) and NM the lowest (64.0). GS, which had one of the highest NPP, also led in terms of NSP, suggesting a potential correlation between these traits. Seed weight per plant (SWP) ranged from 11.38 g in JX to 27.20 g in SX, with SX consistently outperforming the other locations in terms of this trait (*p* < 0.05), which is consistent with its superior performance in terms of PH. The 100-seed weight (HSW) varied significantly across locations, with SX and SD demonstrating the highest values (29.12 g and 28.58 g, respectively), which were significantly greater than those of the other locations (*p* < 0.05). Seed yield (SY) also showed marked geographical variation, with XJ exhibiting the highest yield (266.68 g), significantly surpassing all other locations (*p* < 0.05), whereas NM had the lowest yield (171.21 g).

To analyze the variation patterns in soybean traits across different geographical locations, we performed a principal component analysis (PCA) on a dataset comprising 13 distinct locations (Fig. 1c). The first two principal components (PC1 and PC2) accounted for a cumulative 84.87% of the total variation, with PC1 explaining 55.99% and PC2 contributing an additional 28.88%. This substantial proportion of variance explained by the first two PCs underscores their capacity to capture the majority of the variation in soybean traits across the locations. The PCA biplot revealed distinct clustering patterns among the 13 locations, indicating significant geographical influences on soybean trait expression. NM and JX formed separate clusters on the far right of the plot, suggesting that they have distinct characteristics, particularly along PC1. SX formed an isolated cluster in the lower left quadrant, indicating that unique trait combinations differ from those in all other locations. HB and GS clustered together in the upper left quadrant. JL, HLJ, DQ, and XJ formed a loose cluster in the center right of the plot. LN and SD were grouped in the lower central area, suggesting similarities in their soybean trait profiles. XZ and MDJ clustered in the central area of the plot, implying intermediate trait expression compared with other locations.

Additionally, correlation analysis of the 6 key soybean agronomic traits across 13 locations was conducted (Fig. 1d). The findings of the correlation analysis revealed significant relationships. For instance, PH was positively correlated with all other traits, exhibiting the strongest association with SWP (*r* = 0.63, *p* < 0.001), followed by HSW (*r* = 0.48, *p* < 0.001), NPP (*r* = 0.43, *p* < 0.001), SY (*r* = 0.34, *p* < 0.001), and NSP (*r* = 0.26, *p* < 0.01). HSW had a significant positive correlation with SY (*r* = 0.23, *p* < 0.01) and a strong correlation with SWP (*r* = 0.49, *p* < 0.001). The SY was strongly positively correlated with SWP (*r* = 0.55, *p* < 0.001), NPP (*r* = 0.49, *p* < 0.001), and NSP (*r* = 0.40, *p* < 0.001). Among all the traits, SWP presented the strongest correlations with NPP (*r* = 0.80, *p* < 0.001) and NSP (*r* = 0.73, *p* < 0.001). The highest correlation was observed between NPP and NSP (*r* = 0.87, *p* < 0.001), indicating a robust relationship between these yield components across locations.

### Dynamic changes in rhizosphere soil microorganisms among the sites

**Fig. 2:**
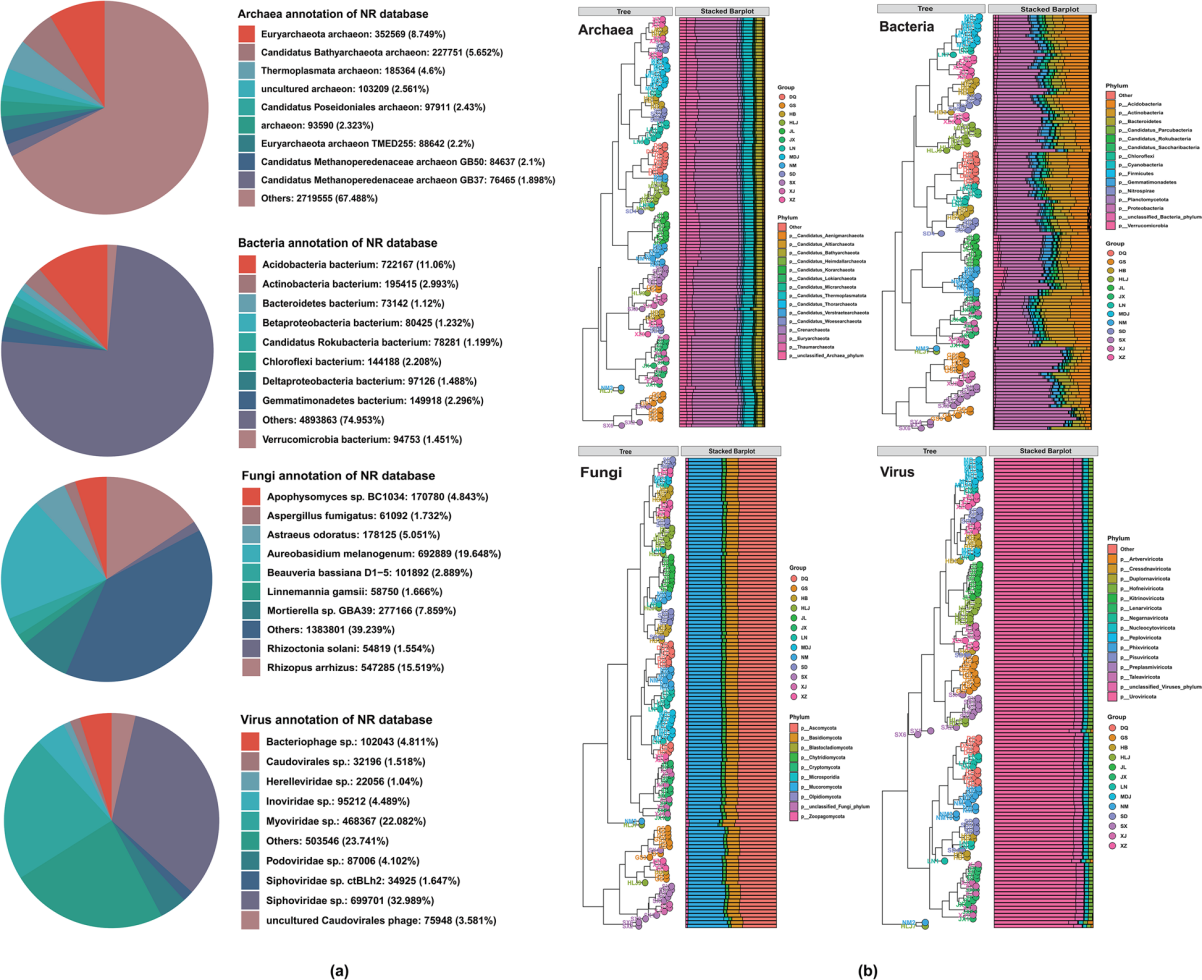
Annotation and classification of rhizosphere soil microorganisms. **a** Nr database annotations and the percentages of the top 10 species were statistically analyzed. **b** Bray‒Curtis similarity-based dendrogram showing the community composition for each compartment at each site. Only mOTUs with an *RA* > 0.1% were considered. For each sample, the community composition (phylum level) is indicated with bar plots

A total of 130 samples were collected from 13 sites in China (10 samples were obtained from each site) to investigate the impact of rhizosphere microorganisms on soybean growth. Metagenomic analysis yielded a total of 920 GB of raw data (Table S1 and S2), which allowed us to identify a total of 43,337 species, including 32,435 bacteria, 2494 archaea, 2118 fungi, and 6290 viruses (Fig. 2a). The species were designated metagenomic operational taxonomic units (mOTUs) for statistical analysis. The rarefaction analysis confirmed that the majority of the rhizosphere microorganisms were successfully collected. The analysis of microbial community structure via average Bray‒Curtis distances revealed clustering by compartments and sites for archaea, bacteria, fungi, and viruses in the root rhizoplane. Only mOTUs with a relative abundance (RA) > 0.1% were considered. At the phylum level, 25 archaeal, 157 bacterial, 10 fungal, and 19 viral phyla were identified from the 43,337 mOTUs in the metagenomic libraries (Fig. 2b).

**Fig. 3:**
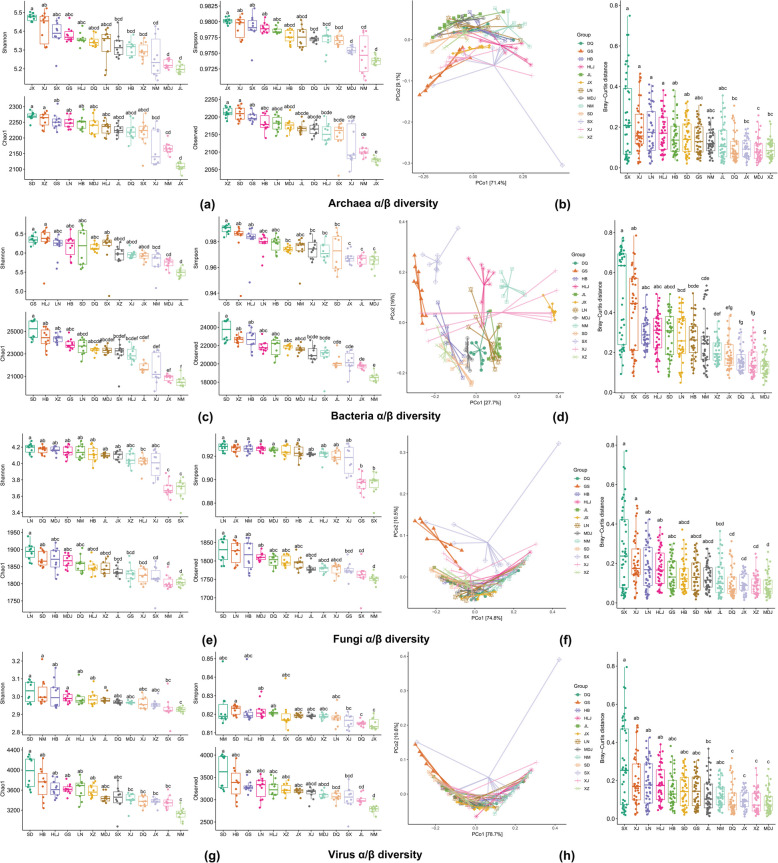
Dynamic changes in rhizosphere soil microorganisms. This figure comprehensively analyzes rhizosphere soil microorganism diversity and community structure. Comparison of rhizosphere soil microorganisms based on alpha-diversity indices (Shannon, Simpson, Chao1, and observed indices) at four kingdoms (Archaea (**a**), Bacteria (**c**), Fungi (**e**), and virus (**g**)). Principal coordinate analysis (PCoA) results based on Bray‒Curtis dissimilarities, which reveal the differences in rhizosphere soil microorganism community structures for each of the four kingdoms (Archaea (**b**), Bacteria (**d**), Fungi (**f**), and virus (**i**)). This PCoA visually represents how the microbial communities are distributed and how their compositions vary within these kingdoms

Alpha-diversity indices (Shannon, Simpson, Chao1, and observed) were calculated for each of the four kingdoms (Archaea, Bacteria, Fungi, and viruses) and revealed significant differences among the 13 sites (Fig. 3a, c, e, g). For the archaea, Shannon diversity was highest at JX, XJ, and SX, whereas the lowest values were observed at JL and MDJ. Similar trends were observed for the Simpson, Chao1, and observed indices, with the highest diversity at JX and XJ and the lowest diversity at JL and MDJ (Fig. 3a). In bacteria, HLJ and GS presented the highest Shannon diversity, whereas JL and MDJ presented the lowest. The Simpson index presented the highest diversity values in GS and SX, whereas the lowest values were revealed by JL and MDJ (Fig. 3c). A similar trend was observed for the Chao1 and observed indices, which peaked at SD, HB, and XZ, whereas the lowest diversity values were recorded at the JX and NM locations. Fungi presented the highest Shannon diversity at LN and DQ, whereas the lowest was at SX and GS. The Simpson index had the highest diversity values in LN and GX, while the lowest values were still in the Shannon index (SX and GS). The Chao1 and observed indices reflected different patterns, with LN and SD showing greater diversity in terms of Chao1 and SD and LN than NM and JX for Chao1 and SX and NM, which presented the lowest diversity (Fig. 3e). For the virus kingdom (Fig. 3g), SD and NM had the highest Shannon diversity, and SX and JX had the lowest. The Simpson, Chao1, and observed indices also showed significant site-specific differences, with NM and SD having higher diversity levels for Simpson, SD, and HB for Chao1. In contrast, DQ and JX for Simpson and JL and NM for Chao1 presented the lowest diversity values. These data highlight the significant variability in microbial communities among the 13 sites, underscoring the influence of site-specific environmental factors on microbial diversity.

Beta-diversity analysis, which employs principal coordinate analysis (PCoA) based on the Bray‒Curtis distance and was assessed via Dunn's Kruskal‒Wallis test, revealed that rhizosphere microorganism community structures were significantly different among the four kingdoms across the 13 sites (Fig. 3b, d, f, h). For all four kingdoms—Archaea, Bacteria, Fungi, and viruses—the PCoA plots demonstrated significant site-specific clustering, indicating distinct community structures among the 13 locations. The first two principal coordinates explained a substantial portion of the variation in community composition for each kingdom, with the first axis accounting for the majority of the variation (71.4% for archaea, 27.7% for bacteria, 74.8% for fungi, and 78.7% for viruses). The Bray‒Curtis distances highlighted consistent trends across all four kingdoms, revealing significant differences in community structure among the sites. For example, sites such as SX and XJ consistently presented distinct microbial communities across all kingdoms, as evidenced by their separation in the PCoA plots and supported by Bray‒Curtis distance analyses. Conversely, sites such as JL and MDJ tended to cluster together, indicating more similar community compositions.

In summary, alpha-diversity indices (Shannon, Simpson, Chao1, and observed) demonstrated significant variations in microbial diversity across the 13 sites, with some locations consistently exhibiting higher or lower diversity across kingdoms. For instance, JX and XJ frequently showed the highest diversity, while JL and MDJ often had the lowest. Beta-diversity analysis using Bray–Curtis distances and PCoA further highlighted distinct community structures among the sites, with unique clustering patterns for different kingdoms. These findings underscore the strong influence of site-specific environmental factors in shaping microbial diversity and community composition.

### Association analysis between the rhizosphere microbiota and soybean phenotype

**Fig. 4:**
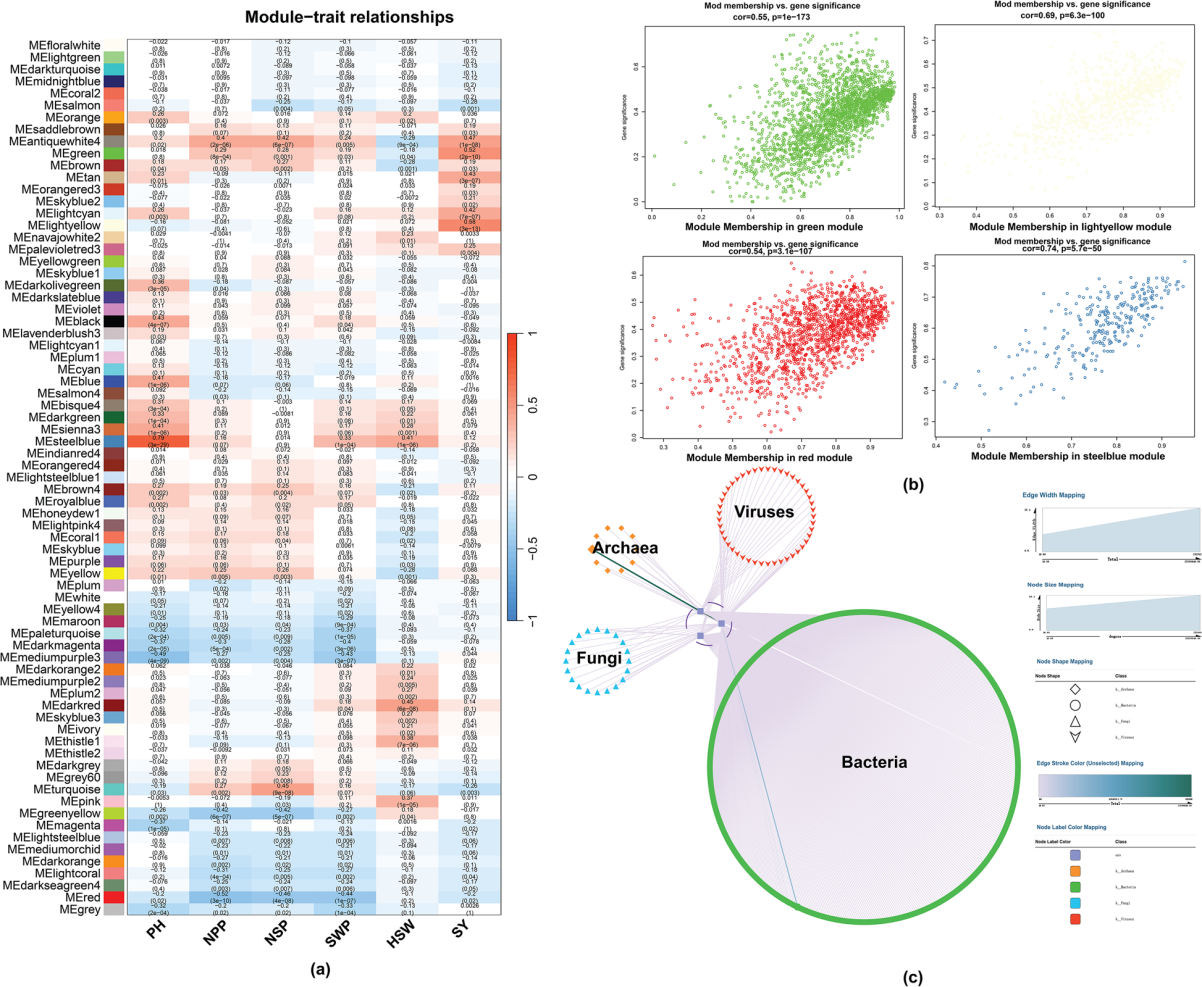
WGCNA of rhizosphere microorganisms associated with soybean phenotypes. **a** Module‒trait associations revealed 73 modules, with the table color-coded by correlation strength according to the color legend. This analysis highlights the relationships between individual modules and key soybean traits (*NSP*, number of seeds per pod; NPP, number of pods per plant (NPP); SWP, seed weight per plant; PH, plant height; HSW, 100-seed weight; SY, seed yield). **b** Four genes were identified with |r|> 0.5 and *P*-value < 0.001, and 556 hub mOTUs were extracted based on *MM* > 0.8 and *GS* > 0.2. These hubs represent key microbial players within each module. **c** The construction of weighted co-expression networks for these microorganisms illustrates the interactions among the hub mOTUs. Specifically, the hub mOTUs associated with Archaea (10), Bacteria (481), Fungi (20), and viruses (45) exhibited strong correlations with the phenotypes PH, NPP, and SY, with |r|> 0.7 and a *p* < 0.05

To investigate the impact of rhizosphere microorganisms on soybean growth and yield, we explored the associations between the relative abundance of rhizosphere microorganisms and the phenotyped characteristics of soybean plants (PH, NPP, NSP, SWP, HSW, and SY) via weighted gene co-expression network analysis (WGCNA) (Fig. 4). The correlation coefficient was calculated for 73 modules in this study; of these, four modules (green, light yellow, red, and steel blue) strongly associated with the phenotypic traits. The correlation was considered significant if the absolute value of the correlation coefficient (|r|) was greater than 0.5 and if the *p* was less than 0.001 (Fig. 4a). The findings revealed a strong positive correlation between the green module and SY (*r* = 0.52, *p* = 2 × 10^−10^), a strong positive correlation between the light-yellow module and SY (*r* = 0.58, p = 2 × 10^−13^), a strong positive correlation between the red module and NPP (*r* = − 0.52, *p* = 2 × 10^−10^), and a strong positive correlation between PH and the steel blue module *(r* = 0.79, *p* = 2 × 10^−29^).

To conduct a detailed analysis of the four modules exhibiting highly significant correlations with the studied traits, we calculated the correlation between gene significance (GS) and module membership (MM) values (Fig. 4b). Modules with |r|> 0.5 and a *p*-value < 0.001 were identified, and 556 hub mOTUs with *MM* > 0.8 and *GS* > 0.2 were extracted (Table S3). The correlation between MM and GS in the green module was highly significant (*r* = 0.55, *p* = 1 × 10^−173^; Fig. 4b, top left). The light-yellow module exhibited a strong positive correlation between MM and GS (*r* = 0.69, *p* = 6.3 × 10^−100^; Fig. 4b, top right). This finding suggests a substantial relationship between module membership and gene significance within this module. In the red module, the correlation between MM and GS was also significant (*r* = 0.54, *p* = 3.1 × 10^−107^; Fig. 4b, bottom left). The steel blue module showed the strongest positive correlation (*r* = 0.74, *p* = 5.7 × 10^−50^; Fig. 4b, bottom right).

An analysis of weighted co-expression networks was conducted to better understand how specific microbial taxa (mOTUs) influence soybean growth parameters (Fig. 4c). We identified 556 hub mOTUs showing significant correlations with key plant metrics: SY, PH, and NPP. These hub mOTUs spanned multiple kingdoms: Bacteria dominated with 481 mOTUs, followed by Fungi (20 mOTUs), Archaea (10 mOTUs), and viruses (45 mOTUs). Bacteria, fungi, and viruses showed strong correlations with all three plant parameters (SY, NPP, and PH), while archaeal mOTUs were specifically associated with SY and PH. The widespread influence of these hub taxa across multiple plant growth parameters suggests their potential utility as biological indicators of soil fertility.

### Distribution and importance of hub microorganisms affecting the soybean phenotype across sites

**Fig. 5:**
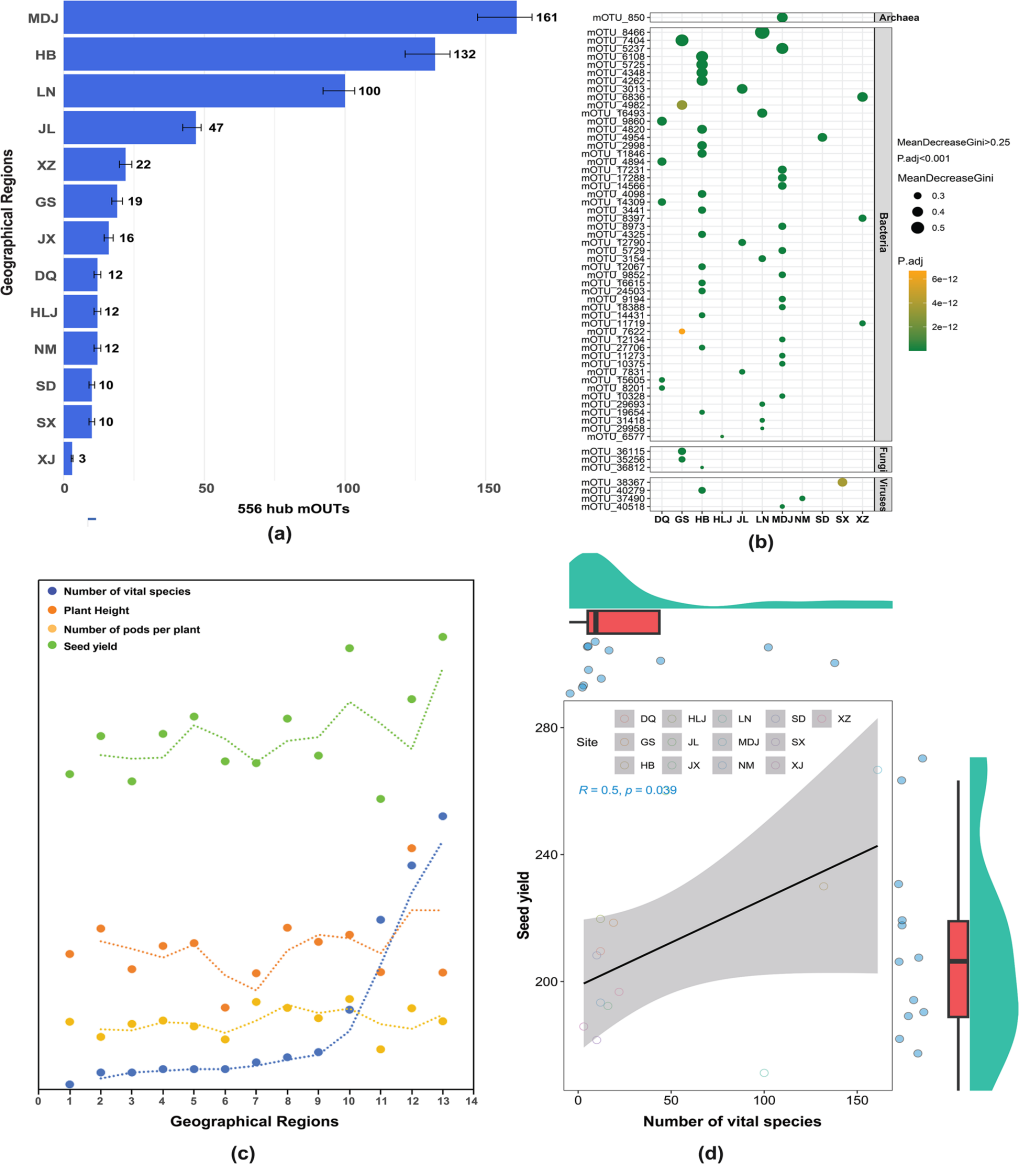
Distribution and impact of hub microorganisms on soybean phenotype across geographical regions. **a** The bar plot illustrates the number of hub microorganisms identified in each of the 13 geographical regions, with error bars representing the variability within regions. The distribution of 556 hub microorganisms is shown across 13 geographical regions in China, with MDJ harboring the highest number (161) and XJ the lowest (3). **b** Random forest analysis revealing key microbial predictors of soybean phenotype. Dot size represents the mean decrease Gini index (0.3–0.5), with larger dots indicating stronger predictive power. Color gradient indicates adjusted *p*-values (ranging from 5e- 12 to 2e- 17), with darker green showing higher significance. Taxonomic classification is shown on the right (Archaea, Bacteria, Fungi). Error bars represent standard deviation from 100 random forest iterations. **c** Relationships between hub microorganism abundance and soybean phenotypic traits across geographical regions (*n* = 13). Yellow dots, number of vital species (mean ± SE); orange dots, plant height (cm, mean ± SE); green dots, number of pods per plant (mean ± SE); blue dots, seed yield (g/plant, mean ± SE). Dotted lines indicate the trend across regions. **d** Linear regression analysis showing the correlation between seed yield and number of vital species (*r* = 0.5, *p* = 0.039). Gray shading represents a 95% confidence interval. Box plots and density plots on margins show the distribution of data points across sites. Each point represents a sampling site, with different symbols indicating geographical regions

The microbial composition and abundance in the soybean rhizosphere varied significantly across different sites. To understand these variations, we focused on the distribution characteristics of the 556 hub mOTUs at each site (Fig. 5, Table S3). Using the random forest method, we assessed the importance of these hub mOTUs at each location. Our analysis revealed distinct enrichment patterns: 161 hub mOTUs in MDJ, 132 in HB, 100 in LN, 47 in JL, 22 in XZ, 19 in GS, 16 in JX, 12 in NM, 12 in HLJ, 12 in DQ, 10 in SX, 10 in SD, and only 3 in XJ (Fig. 5a). These findings highlight the site-specific distribution of key microbial taxa, underscoring their potential roles in enhancing soil fertility and soybean productivity (Fig. 5a). Then, we analyzed the distribution of mOTUs with a mean decrease Gini coefficient > 0.25 and a P.adj < 0.001 at each site. The results indicate that 18 key mOTUs (16 bacteria, 1 fungus, and 1 virus) were significantly enriched in HB, 15 key mOTUs (1 Archaea, 13 Bacteria, and 1 virus) were enriched in MDJ, 6 key mOTUs (all bacteria) were enriched in LN, 5 key mOTUs (all bacteria) were enriched in DQ, 5 key mOTUs (3 bacteria and 2 fungi) were enriched in GS, 3 key mOTUs (all bacteria) were enriched in JL, 3 key mOTUs (all bacteria) were enriched in XZ, 1 key mOTU (bacteria) was enriched in HLJ, 1 key mOTU (bacteria) was enriched in SD, 1 key mOTU (virus) was enriched in NM, and 1 key mOTU (virus) was enriched in SX (Fig. 5b). The figure illustrates the relationships between the number of vital microbial species and three key plant phenotypic traits—plant height, seed yield, and number of pods per plant—across the 13 Chinese geographical locations. A positive correlation was observed between microbial diversity and plant height. However, the relationship between microbial diversity and seed yield was more complex, with an initial positive association followed by a plateau. The association between microbial diversity and the number of pods per plant was less pronounced, suggesting a weaker influence of microbial composition on this particular trait (Fig. 5c). To elucidate the relationship between microbial diversity and seed yield across the 13 geographical locations in China, a significant positive correlation was observed (*r* = 0.5, *p* = 0.039). This finding indicates that an increase in the number of vital microbial species is associated with increased seed yield. The upward trend of the regression line in Fig. 5d visually supports this positive association. However, the distribution of data points around the regression line suggests variability in this relationship across different sites.

### Co-occurrence networks and species composition in rhizosphere microbiota modules

**Fig. 6:**
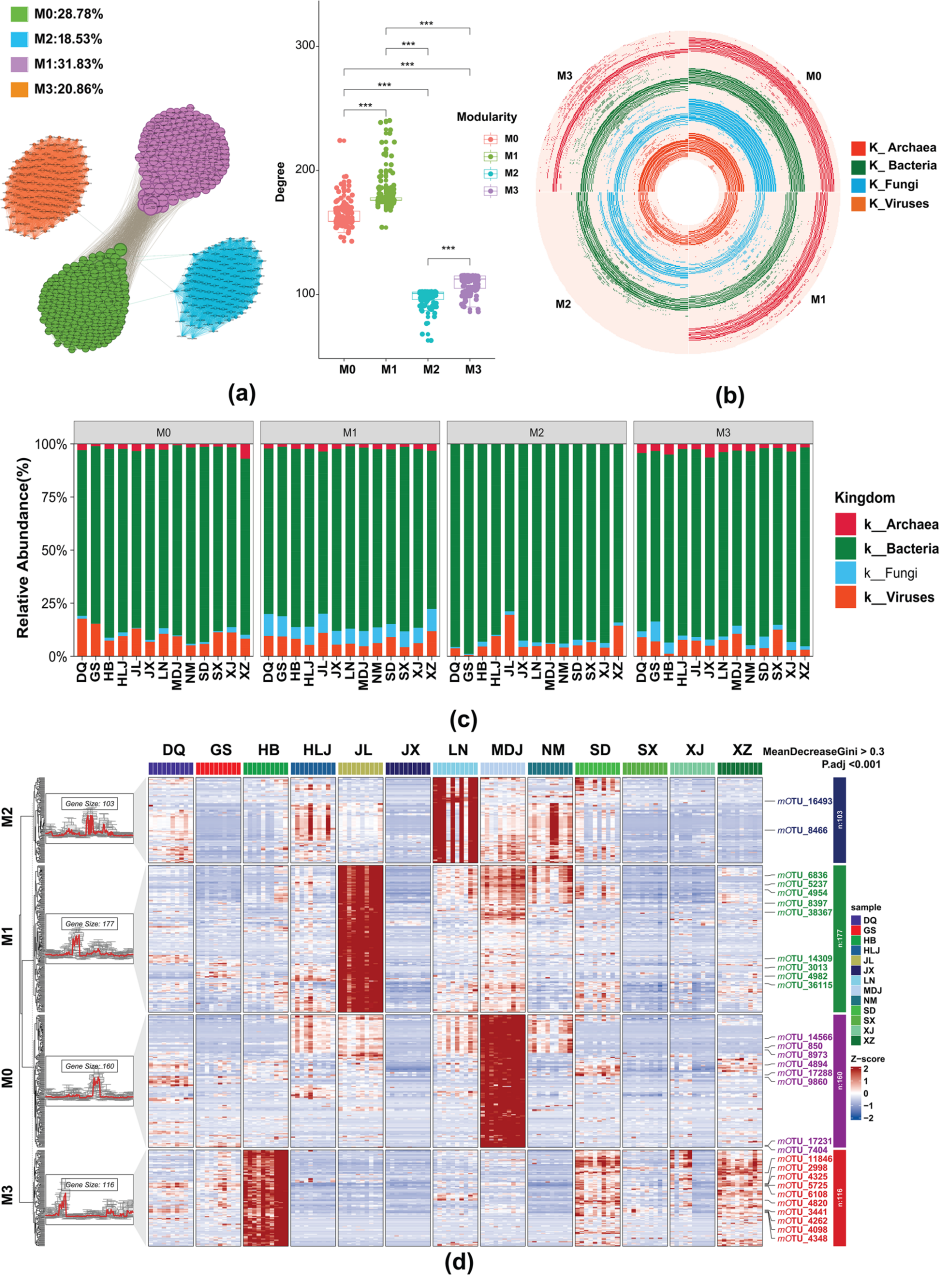
Characteristics of microbial relative abundance changes in the modules. **a** Cooccurrence network clustering of hub rhizosphere microorganisms in soybean, indicating significant differences in the degree coefficients among the four modules (***p < 0.001). **b** Polar circle plots depicting species composition within each module, where M0, M1, and M3 contain members from four kingdoms (Archaea, Bacteria, Fungi, and viruses), but M2 lacks Archaea. **c** Kingdom-level distribution of microbial communities across different sites for each module, showing the relative abundances of Archaea, Bacteria, Fungi, and viruses. **d** Clustering heatmap of 556 hub mOTUs within the four modules, with M0 comprising 160 hub mOTUs, M1 comprising 177, M2 comprising 103, and M3 comprising 116. The 29 key hub mOTUs were identified via the criteria of mean decrease Gini > 0.3 and P.adj < 0.001: M0 (8/160), M1 (9/177), M2 (2/103), and M3 (10/116). The heatmap highlights the specific hub mOTUs with the highest abundance in particular locations, illustrating the distribution patterns and ecological significance of these microbial communities

We constructed co-occurrence networks using 556 mOTUs to elucidate potential interaction patterns within rhizosphere microbial communities associated with soybean across 13 geographically distinct locations in China (Fig. 6, Table S3). The number of linkages, indicative of the strength of potential microbial interactions, was highest in module M1, followed by M0 and M3, with the lowest number observed in M2. These four modules corresponded to the color modules identified by WGCNA: M0 (light yellow: 28.78%), M1 (green: 31.83%), M2 (red: 18.53%), and M3 (steel blue: 20.86%) (Fig. 6a). The network degree, which quantifies the significance of nodes within the network, exhibited the following pattern: M1 > M0 > M3 > M2. Significant differences in the degree coefficients among the four modules were noted (***p < 0.001), underscoring the varying importance and interaction density of microbial taxa within each module. To gain a comprehensive understanding of the composition of each module, we evaluated the relative abundance of the hub rhizosphere microbial communities at each site. The analysis revealed that three modules (M0, M1, and M3) contained members from four kingdoms: Archaea, Bacteria, Fungi, and viruses. In contrast, module M2 lacked representation from the Archaea kingdom (Fig. 6b, c). This detailed composition analysis indicated that bacteria dominated the microbial communities across all modules and sites, consistently resulting in the highest relative abundance (Fig. 6c). The presence of fungi and viruses varied across modules and locations, with M1 and M0 showing relatively higher proportions of these kingdoms than M2 and M3 (Fig. 6c). The absence of Archaea in M2 suggests that distinct ecological niches or environmental conditions could influence the microbial community structure and interactions within this module.

To elucidate the distribution patterns and interactions of the hub microbial communities within the rhizosphere, we constructed a clustering heatmap of 556 hub mOTUs across the four identified modules. The heatmap revealed that module M0 comprised 160 hub mOTUs, M1 comprised 177, M2 comprised 103, and M3 comprised 116 (Fig. 6d). Using the criteria of a mean decrease Gini coefficient > 0.3 and P.adj < 0.001, we identified a total of 29 hub mOTUs, which were categorized as follows: M0 (8 out of 160), M1 (9 out of 177), M2 (2 out of 103), and M3 (10 out of 116). In module M0, MDJ presented the highest abundance of hub mOTUs, with notable mOTUs, including mOTU_14566 (Bacteria), mOTU_850 (Archaea), mOTU_8973 (Bacteria), mOTU_4894 (Bacteria), mOTU_17288 (Bacteria), and mOTU_9860 (Bacteria). Within module M1, JL displayed the greatest abundance, highlighted by mOTUs such as mOTU_14309 (Bacteria), mOTU_3013 (Bacteria), mOTU_36115 (Fungi), mOTU_38367 (virus), mOTU_4954 (Bacteria), mOTU_4982 (Bacteria), mOTU_5237 (Bacteria), mOTU_6836 (Bacteria), and mOTU_8397 (Bacteria). Module M2 had the highest prevalence in LN, as indicated by the presence of mOTU_16493 (bacteria) and mOTU_8466 (bacteria). In module M3, HBs presented the maximum abundance, represented by mOTU_11846 (Bacteria), mOTU_2998 (Bacteria), mOTU_3441 (Bacteria), mOTU_4098 (Bacteria), mOTU_4262 (Bacteria), mOTU_4325 (Bacteria), mOTU_4348 (Bacteria), mOTU_4820 (Bacteria), mOTU_5725 (Bacteria), and mOTU_6108 (Bacteria).

**Fig. 7:**
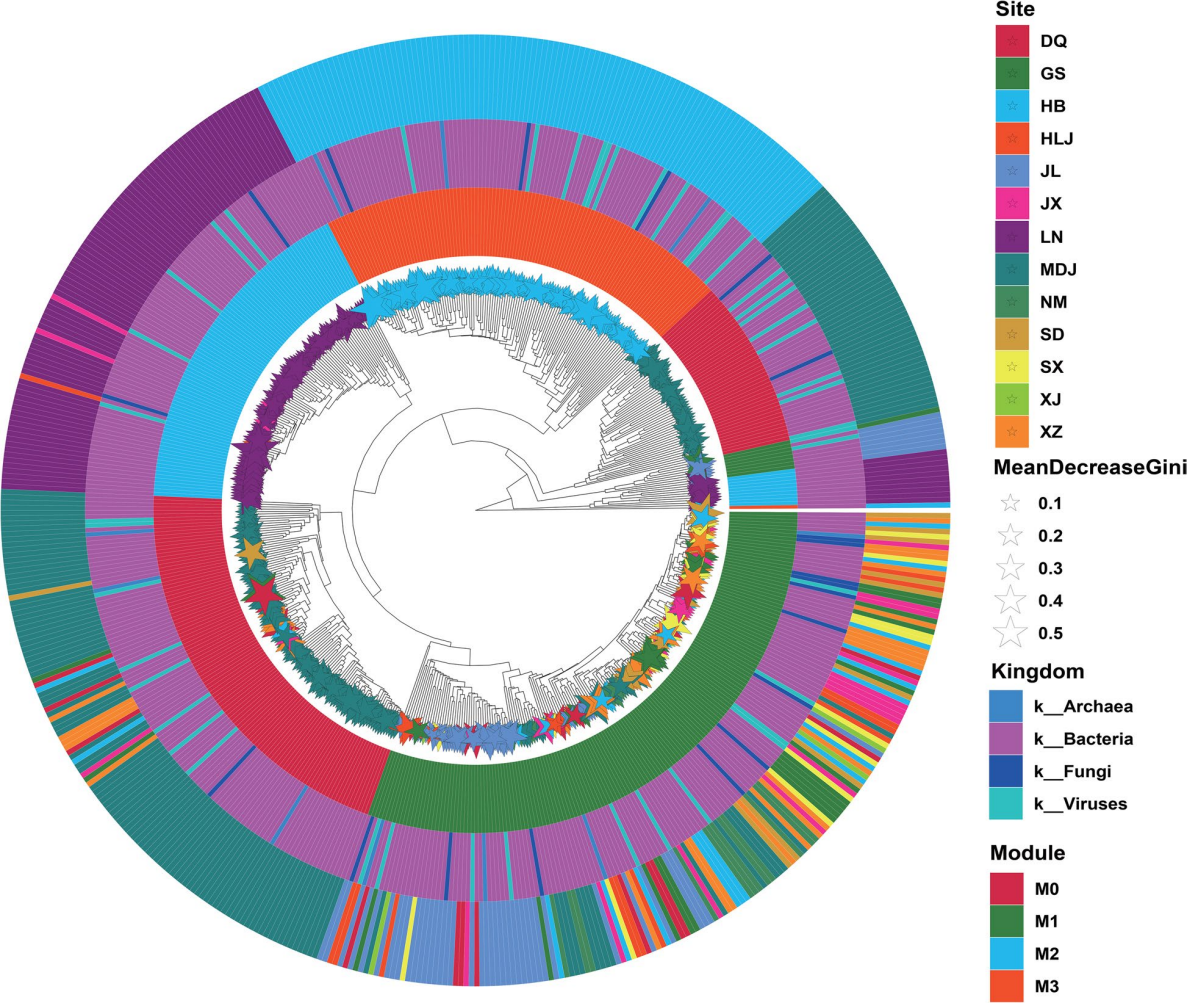
Evolutionary relationship tree of hub microorganisms. The phylogenetic tree, constructed via the unweighted pair group method with arithmetic mean (UPGMA), encompasses 556 hub mOTUs. From the inside to the outside, the size of the star nodes in the innermost ring indicates the importance of each microorganism as determined by random forest analysis. The second ring depicts the clustering of microorganisms into four distinct modules: M0, M1, M2, and M3. The third ring illustrates the taxonomic classification of these microorganisms at the kingdom level, identifying members from Archaea, Bacteria, Fungi, and viruses. The outermost ring represents the geographical sampling sites, showing the distribution of these key microorganisms across different locations

To elucidate the evolutionary relationships among the central microorganisms, we constructed a phylogenetic tree via the unweighted pair group method with arithmetic mean (UPGMA) approach (Fig. 7). This phylogenetic tree included 556 hub microorganisms (Table S4). The innermost ring indicates the importance of each microorganism, with star node sizes representing their significance as determined by a random forest model. The second ring displays the clustering into four modules: M0, M1, M2, and M3. The third ring categorizes the microorganisms at the kingdom level, encompassing Archaea, Bacteria, Fungi, and viruses. The outermost ring highlights the geographical locations from which the samples were collected, providing a comprehensive view of the distribution and evolutionary relationships of these key microbial taxa across different sites.

### Metabolic network analysis of hub rhizosphere microorganisms in CNPS cycling

To elucidate the underlying mechanisms of the hub rhizosphere microbiota, we compared the metagenomic data against several functional gene annotation databases (MCycDB, NCycDB, PCyCDB, and SCycDB) (Table S5). Our analysis identified 151 key orthologs (KOs): 70 associated with the carbon cycle, 16 with the nitrogen cycle, 51 with the phosphorus cycle, and 14 with the sulfur cycle. We investigated the correlation between the rhizosphere microbiota and CNPS (carbon, nitrogen, phosphorus, and sulfur) functional genes via Pearson correlation analysis, with criteria set at |r|> 0.7 and *p* < 0.001 (Table S5). This revealed significant positive correlations between 254 mOTUs and 115 KOs. Specifically, 65 mOTUs within module M0 displayed strong positive correlations with 100 KOs (carbon: 48, nitrogen: 15, phosphorus: 32, sulfur: 5), 174 mOTUs within module M1 correlated with 41 KOs (carbon: 19, nitrogen: 3, phosphorus: 12, sulfur: 6), 10 mOTUs within module M2 correlated with 7 KOs (carbon: 4, nitrogen: 1, sulfur: 2), and 5 mOTUs within module M3 correlated with 2 KOs (carbon: 2). Interestingly, the findings also indicated multiple significant correlations were observed across different biogeochemical cycles. For the carbon cycle, several mOTUs from different modules, including those from M0 (mOTU_11306) and M2 (mOTU_7964, mOTU_13082, mOTU_8056, and mOTU_8451), showed strong negative correlations with the carbon fixation-related KO K14468, with correlation coefficients ranging from − 0.70 to − 0.75. In the nitrogen cycle, fungi-associated mOTUs from module M1 (mOTU_36115) demonstrated positive correlations with both nitrate reduction (K00370, *r* = 0.704986) and nitrite reduction (K00368, *r* = 0.71), while bacterial mOTU_28710 from M0 correlated positively with nitrate reduction (K00371, *r* = 0.7). For phosphorus cycling, viral mOTU_39049 from M0 showed positive correlation with organic P mineralization (K06164, *r* = 0.7), and bacterial mOTU_7404 from M0 correlated with regulatory functions (K02039, *r* = 0.7). Notably, sulfur oxidation processes exhibited consistent positive correlations across multiple bacterial mOTUs from module M1, with mOTU_22692, mOTU_8357, and mOTU_8489 correlating with K17725 (*r* ranging from 0.70 to 0.71), while mOTU_26725 and mOTU_5205 showed strong positive correlations with K16952 (*r* = 0.71 and 0.72, respectively). Additional notable correlations can be found in Table S5.

**Fig. 8:**
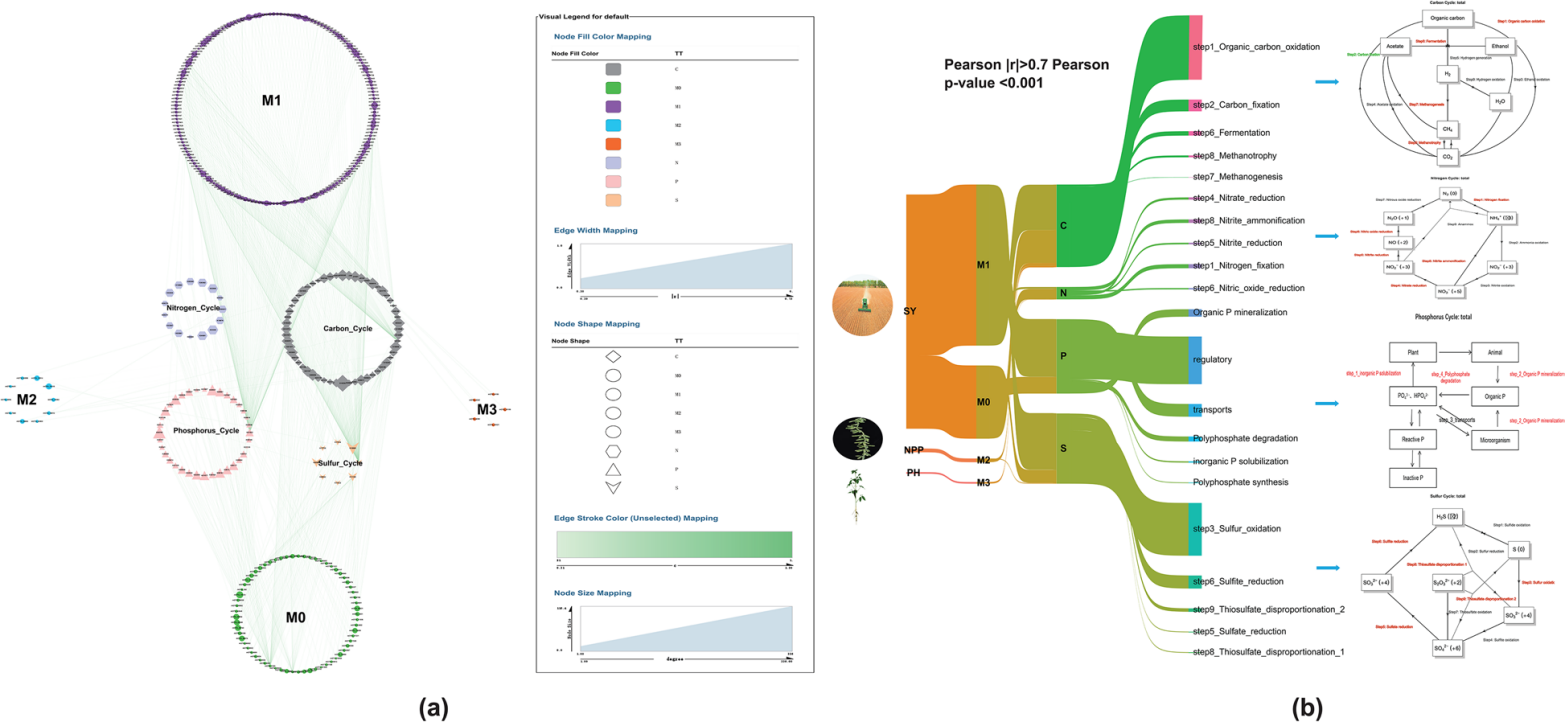
Correlation networks and pathway mapping of CNPS-related genes in modular communities. **a** Correlation network showing relationships between modular microbial taxa (mOTUs) and key orthologs (KOs) involved in carbon, nitrogen, phosphorus, and sulfur cycles. Line thickness represents correlation strength (|r|> 0.7, *p* < 0.001), and node size indicates relative abundance of mOTUs or KOs. **b** Sankey diagram illustrating the interconnections between three soybean phenotypes (NPP, number of pods per plant; PH, plant height; SY, seed yield), microbial modules (M0–M3), and functional gene pathways in CNPS cycling. The hierarchical flow (left to right) demonstrates the linkages from plant phenotypes through microbial community structure to metabolic functions. Red connections indicate positive correlations, while green connections represent negative correlations

Additionally, notable correlations between the functional gene profiles and the four modules within the CNPS pathway were revealed (Fig. 8b). Within the carbon cycle, functional steps 1 (organic carbon oxidation, encompassing 39 KOs), 3 (fermentation, involving 8 KOs), 7 (metabolism, represented by 1 KO), and 8 (methanotrophy, comprising 3 KOs) are significantly enriched, demonstrating a positive association with hub microorganisms. In contrast, step 2 (carbon fixation), which included 5 KOs, was enriched, with a significant negative correlation with the hub microorganisms. Hub microorganisms also facilitate the nitrogen cycle. Specifically, steps involving nitrogen fixation (step 1, 3 KOs), nitrate reduction (step 4, 4 KOs), nitrite reduction (step 5, 2 KOs), nitric oxide reduction (step 6, 2 KOs), and nitrite ammonification (step 8, 4 KOs) enriched 15 KOs with significant positive correlations. These findings indicate that hub microorganisms promote nitrogen utilization by enhancing both nitrogen fixation and denitrification processes. In the phosphorus cycle, steps involving inorganic P solubilization (step 1, 3 KOs), organic P mineralization (step 2, 11 KOs), and polyphosphate degradation (step 4, 6 KOs) enriched 20 KOs with significant positive correlations. These results indicate that the hub microorganisms promote phosphorus utilization. In the sulfur cycle, the hub microorganisms significantly enriched 10 KOs, including sulfur oxidation (step 3, 2 KOs), sulfate reduction (step 5, 1 KO), sulfite reduction (step 6, 2 KOs), and thiosulfate disproportionation (steps 8 and 9, 3 KOs). These findings suggest that hub microorganisms promote sulfur utilization by enhancing desulfurization and sulfurization processes.

## Discussion

### Geographical and microbial influences on soybean agronomic performance

The geographical distribution of the study sites and their corresponding microbial communities provide valuable insights into the interactions between environmental factors and soybean agronomic performance. The observed variations in microbial community compositions highlight the significant influence of local environmental conditions on the predominance of specific microbial kingdoms. This is particularly evident in the consistent dominance of bacteria at the JX site and the relatively low percentages of viruses across all locations. Recent studies have emphasized the crucial role of rhizosphere microbiomes in enhancing crop productivity and stress resilience, with evidence suggesting that variations in rhizosphere microbes can significantly affect soybean yield [48]. However, it is essential to note that geographical variability may mask the effects of other factors, such as soil type, fertilizer application, and cropping history, which could independently influence microbial profiles. Fertilizer inputs are known to alter microbial community dynamics, potentially confounding the relationship between rhizosphere characteristics and soybean traits [2]. These aspects should be carefully disentangled in future research.

The geographical variability in soybean traits across the 13 locations reveals the substantial impact of regional environmental factors on plant development and productivity. The significant differences in traits such as plant height, number of pods per plant, number of seeds per pod, seed weight per plant, 100-seed weight, and seed yield underscore the adaptive responses of soybean to diverse environmental conditions [49]. Locations such as SX, which have strong effects on multiple traits, are likely to benefit from favorable growing conditions or effective local agricultural practices. Interestingly, these findings partially align with previous studies on genotype‒environment interactions in other crops, though contrasting patterns of trait expression in specific environments suggest that local soil management practices could be equally influential [4]. Further investigation is warranted to establish whether similar patterns hold across broader geographical scales.

Our extensive metagenomic analysis of rhizosphere microorganisms from 13 diverse sites in China revealed a rich tapestry of microbial diversity and site-specific signatures. The identification of 43,337 species, including bacteria, archaea, fungi, and viruses, underscores the complexity of the microbial landscape in soybean rhizospheres. Recent research highlights the crucial role of microbial diversity in plant health and productivity, with site-specific clustering of microbial communities providing insights into their impact on soybean growth [50, 51]. The identification of 25 archaeal, 157 bacterial, 10 fungal, and 19 viral phyla aligns with contemporary studies emphasizing the importance of microbial community composition and its functional potential in agricultural settings [52–54]. This detailed microbial profiling demonstrates how variations in microbial communities can significantly influence plant resilience and productivity, offering valuable insights for optimizing agricultural practices.

### Microbial diversity and community structure in soybean rhizospheres

Understanding the diversity of microbial communities within soybean rhizospheres across different geographical locations is crucial for deciphering the interplay between environmental factors and microbial dynamics. Recent analyses of alpha-diversity indices, including Shannon, Simpson, Chao1, and observed metrics, revealed substantial variation in microbial diversity among the 13 study sites, with distinct patterns observed across archaea, bacteria, fungi, and viruses. These variations are consistent with findings from other crops, where microbial diversity was strongly influenced by local soil nutrient profiles, pH, and moisture levels [55]. However, it remains unclear to what extent crop management practices, including pesticide and herbicide use, could have shaped these microbial community structures. Beta-diversity analysis via PCoA and Bray‒Curtis distance confirmed significant site-specific differences in microbial community structure. Sites such as SX and XJ presented unique microbial profiles, whereas JL and MDJ clustered together, indicating more similar community compositions. While these results align with prior studies demonstrating the regional specificity of microbial communities, they also highlight potential limitations. For instance, local agricultural practices such as the application of organic versus chemical fertilizers might have introduced variability independent of natural environmental factors [56]. Addressing these confounders in future research is crucial for a holistic understanding of microbial impacts on crop productivity.

The investigation of the impact of rhizosphere microorganisms on soybean growth and yield through WGCNA provides a nuanced understanding of how microbial communities influence crop performance. The strong correlations identified between specific microbial modules and soybean phenotypic traits underscore the critical role of the rhizosphere microbiome in shaping plant development and productivity. Recent studies have demonstrated the profound impact of rhizosphere microorganisms on crop performance. For example, the positive correlations between the green and light-yellow modules and soybean SY align with recent findings [57, 58], which reported similar associations in other crops, suggesting that these microbial communities increase yield potential by influencing nutrient availability and plant growth regulation. The significant relationship between the steel blue module and pH further supports the role of specific microbial communities in optimizing plant stature, as shown in recent research highlighting the interplay between soil microorganisms and plant growth regulators [58, 59].

Additionally, the analysis of module membership and gene significance confirmed that the green and light-yellow modules, which presented high positive correlations, contained microbial mOTUs with substantial influence on yield traits. This finding is consistent with the work of Moretti et al. [60], who identified similar patterns of microbial influence on agronomic traits, suggesting that these modules may be key players in the regulatory networks affecting soybean productivity. Importantly, the identification of 556 hub mOTUs with significant correlations with SY, PH, and NPP highlights the potential of these microorganisms as biomarkers for soil fertility and crop productivity. Previous investigations have emphasized the utility of microbial biomarkers in predicting plant performance and optimizing soil management practices [61, 62]. The substantial presence of bacteria among the identified mOTUs underscores the central role of bacterial communities in influencing key agronomic traits, which is supported by recent literature on the beneficial effects of bacterial diversity on crop yield and resilience [63, 64]. Furthermore, the significant correlations with fungal and viral mOTUs highlight the complex interactions within the rhizosphere microbiome that contribute to soybean growth and productivity. Recent research by Adedayo et al. [52] and Liu et al. [17] has shown that fungal communities can impact plant health and yield through mechanisms such as symbiosis and pathogen suppression, whereas viral interactions often play a role in modulating plant stress responses [65–67].

### Site-specific microbial enrichment and its implications for soil health and crop productivity

The varied enrichment of key microbial taxa across sites underscores the complexity of microbial ecosystems and their functional roles. For example, the high number of hub mOTUs in locations such as MDJ and HB suggests that these areas may have favorable conditions that support diverse microbial communities. This diversity is essential for maintaining soil health and promoting robust plant growth [68]. The significant site-specific enrichment of key mOTUs, including bacteria, fungi, and viruses, could play crucial roles in plant health and productivity. For example, the significant enrichment of bacterial mOTUs in HB and MDJ aligns with studies showing that bacteria are vital for nitrogen fixation [69] and disease suppression [70]. Nevertheless, the specificity of these enriched taxa to particular environments suggests that universal application of findings across locations may be challenging. Previous studies have highlighted the importance of environmental context in determining the efficacy of microbial-based interventions [71]. This variability underscores the need to consider regional adaptations when designing agricultural practices aimed at enhancing microbial diversity and function.

The co-occurrence networks and detailed species composition analysis underscore the critical role of microbial interactions in shaping rhizosphere dynamics and influencing soybean agronomic traits. By understanding these complex interactions, we can develop targeted strategies to manipulate microbial communities for improved crop performance and resilience. The construction of co-occurrence networks from 556 mOTUs in this study provided a comprehensive understanding of the interaction patterns within rhizosphere microbial communities across diverse geographical locations. The dominance of bacteria in all modules aligns with previous findings that bacteria are pivotal in nutrient transformation and disease suppression in the rhizosphere [34]. In contrast, the absence of Archaea in one of the modules, module M2, could indicate niche-specific factors or environmental constraints that limit their presence, reflecting the need for tailored agricultural practices to support diverse microbial communities.

### Functional insights into rhizosphere microbiota and their role in CNPS cycles

The exploration of the rhizosphere microbiota and its functional roles within the CNPS cycles revealed elaborate and significant microbial contributions to nutrient cycling in the soil. By leveraging advanced metagenomic analyses and functional gene annotation databases, our study identified 151 KOs associated with these biogeochemical cycles. Recent research has emphasized the importance of such microbial functional studies in understanding and enhancing agricultural sustainability [72]. However, discrepancies between our findings and those of earlier studies, particularly regarding the dominance of specific microbial pathways in certain locations, may reflect methodological differences or unaccounted confounders such as soil amendments or irrigation practices [73]. Additionally, the strong correlations between specific microbial mOTUs and functional genes suggest that hub microorganisms play critical roles in the CNPS pathways. For example, the positive correlations between module M0 mOTUs and carbon cycle genes indicate substantial involvement in organic carbon oxidation and methanotrophy. This aligns with recent findings that highlight the role of specific microbial taxa in carbon sequestration and greenhouse gas mitigation [74].

Our findings demonstrate that in the nitrogen cycle, hub microorganisms significantly increase nitrogen fixation and denitrification processes. This is particularly relevant given the ongoing challenges of nitrogen management in agricultural systems, where efficient nitrogen utilization can mitigate environmental impacts. The enrichment of KOs related to nitrate and nitrite reduction further underscores the potential of these microorganisms to improve nitrogen efficiency and reduce nitrogen losses through leaching or volatilization. Phosphorus availability is a critical factor for plant growth, and our study highlights the role of hub microorganisms in phosphorus solubilization and mineralization. The positive correlations with KOs involved in these processes suggest that these microorganisms can increase phosphorus availability to plants, supporting recent research on microbial phosphorus cycling in agricultural soils [75]. The findings that specific mOTUs are associated with polyphosphate degradation also offer insights into potential strategies for improving phosphorus use efficiency in crops. In the sulfur cycle, the involvement of hub microorganisms in sulfur oxidation and reduction processes indicates their essential role in sulfur transformation. This phenomenon has significant implications for soil health and crop nutrition, as sulfur is a vital nutrient for plant growth and development. Recent studies have shown that enhancing sulfur cycling through microbial activity can improve crop resilience to environmental stressors [18].

Despite these promising insights, it is important to acknowledge the inherent limitations of metagenomic approaches, which may not fully capture functional gene expression under field conditions. Integrating transcriptomic or proteomic analyses could provide a more comprehensive understanding of microbial functionality [76].

## Conclusion

In this study, we conducted a comprehensive analysis of rhizosphere microbial communities associated with soybean crops across 13 distinct geographical locations in China. Our findings highlight significant variations in microbial diversity and community composition driven by site-specific environmental factors. High-throughput metagenomic sequencing allowed us to identify a broad spectrum of microbial taxa, revealing intricate relationships between microbial ecology and soybean agronomic traits. Principal coordinate analysis (PCoA) and correlation studies demonstrated that microbial communities exhibit distinct clustering patterns and significant associations with key soybean traits, such as plant height, number of pods per plant, seed weight per plant, and seed yield. These results underscore the critical role of rhizosphere microorganisms in influencing soybean growth and productivity. Our investigation into the functional potential of these microbial communities revealed their essential roles in nutrient cycling and plant health. Specific microbial taxa were found to be pivotal in enhancing soybean yield, suggesting potential targets for microbial-based agricultural interventions. The insights gained from this study emphasize the importance of integrating microbial community dynamics into crop management strategies. By understanding the interactions between soil microorganisms and plant traits, we can develop tailored agricultural practices that leverage beneficial microbial functions to optimize crop resilience and yield. This approach paves the way for sustainable agricultural innovations that can enhance food security and environmental health. In conclusion, this research provides a foundational understanding of soybean rhizosphere microbiomes and their impact on crop performance. It highlights the potential of harnessing microbial diversity for improved agricultural outcomes, advocating for a more holistic and ecologically informed approach to modern farming practices.

Future research should concentrate on three main areas: First, field studies should assess the effectiveness of identified beneficial bacteria as bioinoculants, namely their persistence and colonization efficiency under varied agricultural circumstances. Second, research on microbiome modification should investigate how various agricultural methods affect the formation and maintenance of beneficial microbial networks. Third, mechanistic research should explore the molecular interactions between identified hub microbes and plants, with an emphasis on maximizing their favorable impacts on crop resilience and productivity.

## Supplementary Information

Additional file 1: Table S1. Metagenomic analysis yielded a total of 920 gigabytes of raw data. Table S2. Summary of metagenomic sequence statistics. Table S3. Distribution of hub mOTUs across soybean rhizosphere sites. Table S4. Data for phylogenetic tree of hub microorganisms in soybean rhizosphere. Table S5. Functional gene annotations of hub microbiota in soybean rhizosphere with CNPS.

## Data Availability

Sequence data were deposited into NCBI Sequence Read Archive (SRA) under the accession number PRJNA1128828 (https://www.ncbi.nlm.nih.gov/bioproject/?term=PRJNA1128828) . All data generated or analyzed during this study are included in this published article (and its supplementary information files).
